# Crystal structures and anti­oxidant capacity of (*E*)-5-benz­yloxy-2-{[(4-chloro­phen­yl)imino]­meth­yl}phenol and (*E*)-5-benz­yloxy-2-({[2-(1*H*-indol-3-yl)eth­yl]iminium­yl}meth­yl)phenolate

**DOI:** 10.1107/S2056989018003687

**Published:** 2018-03-09

**Authors:** Nadir Ghichi, Chawki Bensouici, Ali Benboudiaf, Yacine DJebli, Hocine Merazig

**Affiliations:** a Unite of Research CHEMS, University of Constantine 1, Algeria; b The Centre of Research in Biotechnology, Constantine, Algeria; c Laboratory of Material Chemistry, University of Constantine 1, Algeria

**Keywords:** crystal structure, Schiff base, DPPH, CUPRAC, anti­oxidant capacity, charge-assisted hydrogen bonding, *X*—H⋯π inter­actions

## Abstract

The title Schiff base compounds, C_20_H_16_ClNO_2_ (I) and C_24_H_22_N_2_O_2_ (II), were synthesized *via* the condensation reaction of 2-amino-4-chloro­phenol for (I), and 2-(2,3-di­hydro-1*H*-indol-3-yl)ethan-1-amine for (II), with 4-benz­yloxy-2-hy­droxy­benzaldehyde. In both compounds, the configuration about the C=N imine bond is *E*. Neither mol­ecule is planar. In (I), the central benzene ring makes dihedral angles of 49.91 (12) and 53.52 (11)° with the outer phenyl and chloro­phenyl rings, respectively. In (II), the central benzene ring makes dihedral angles of 89.59 (9) and 72.27 (7)°, respectively, with the outer phenyl ring and the mean plane of the indole ring system (r.m.s. deviation = 0.011 Å). In both compounds there is an intra­molecular hydrogen bond forming an *S*(6) ring motif; an O—H⋯O hydrogen bond in (I), but a charge-assisted N^+^—H⋯O^−^ hydrogen bond in (II). In the crystal of (I), mol­ecules are linked by C—H⋯π inter­actions, forming slabs parallel to plane (001). In the crystal of (II), mol­ecules are linked by pairs of N—H⋯O hydrogen bonds, forming inversion dimers. The dimers are linked by C—H⋯O hydrogen bonds, C—H⋯π inter­actions and a weak N—H⋯π inter­action, forming columns propagating along the *a*-axis direction. The anti­oxidant capacity of the synthesized compounds was determined by cupric reducing anti­oxidant capacity (CUPRAC) for compound (I) and by 2,2-picrylhydrazyl hydrate (DPPH) for compound (II).

## Abstract 2

The title Schiff base compounds, (I) and (II), were synthesized *via* the condensation reaction of 2-amino-4-chloro­phenol for (I), and 2-(2,3-di­hydro-1*H*-indol-3-yl)ethan-1-amine for (II), with 4-benz­yloxy-2-hy­droxy­benzaldehyde. In both compounds, there is an intra­molecular hydrogen bond forming an *S*(6) ring motif; an O—H⋯O hydrogen bond in (I), but a charge-assisted N^+^—H⋯O^−^ hydrogen bond in (II).

## Chemical context   

Schiff bases of the general type *RR*′C=N*R′′* exhibit a wide structural diversity and have found a wide range of applications (Jia & Li, 2015 ▸). Schiff base derivatives are a biologically versatile class of compounds possessing diverse activities, such as anti-oxidant (Haribabu *et al.*, 2015 ▸, 2016 ▸), anti-inflammatory (Alam *et al.*, 2012 ▸), anti­anxiety, anti­depressant (Jubie *et al.*, 2011 ▸), anti-tumour, anti­bacterial, and fungicidal properties (Refat *et al.*, 2008 ▸; Kannan & Ramesh, 2006 ▸). They can be used as potential materials for optical memory and switch devices (Zhao *et al.*, 2007 ▸). Besides their biological applications, many Schiff bases also reversibly bind with oxygen, coordinate with and show fluorescent variability with metals, exhibiting photo-chromism and/or thermochromism, and have been used as catalysts, pigments and dyes, corrosion inhibitors, polymer stabilizers, or precursors in the formation of nanoparticles (Gupta & Sutar, 2008 ▸; Gupta *et al.*, 2009 ▸; Mishra *et al.*, 2012 ▸). The common structural feature of these compounds is the presence of an azomethine group linked by an *η-*methyl­ene bridge, which can act as hydrogen-bond acceptors. In view of this inter­est we have synthesized the title compounds, (I) and (II), and report herein on their crystal structures. The ^1^H NMR spectra revealed the presence of an imino group (N=CH) in the range δ = 8.5–8.6 p.p.m. Cupric reducing anti­oxidant capacity (CUPRAC) of (I) was estimated, and the anti­oxidant capacity of compound (II) was determined by *in vitro* 2,2-diphenyl-1-picrylhydrazil hydrate (DPPH) radical scavenging.
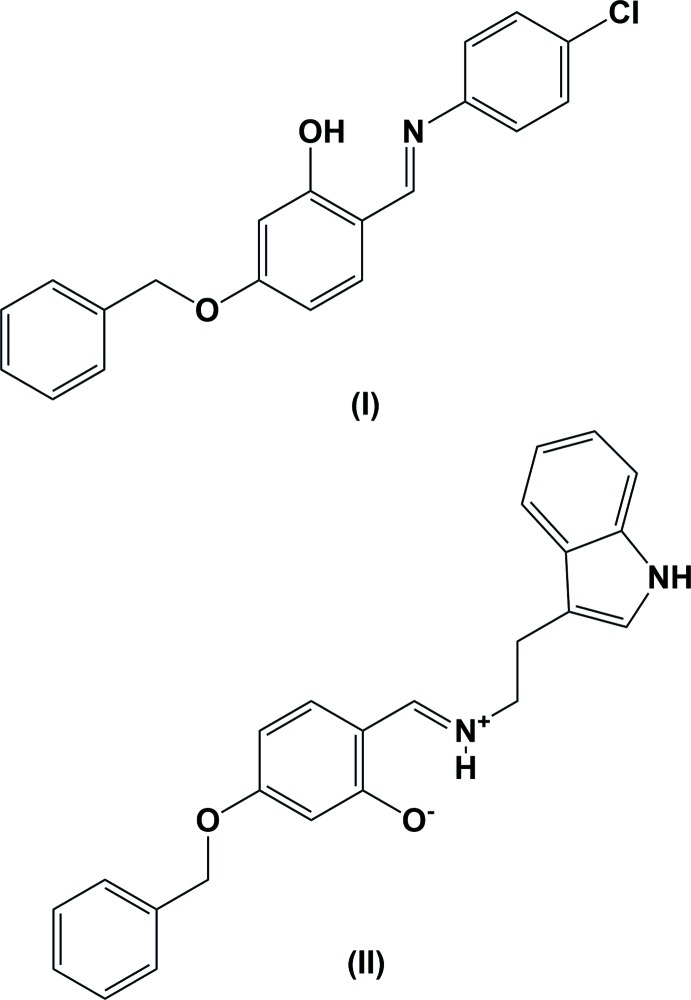


### Structural commentary   

**Figure 1:**
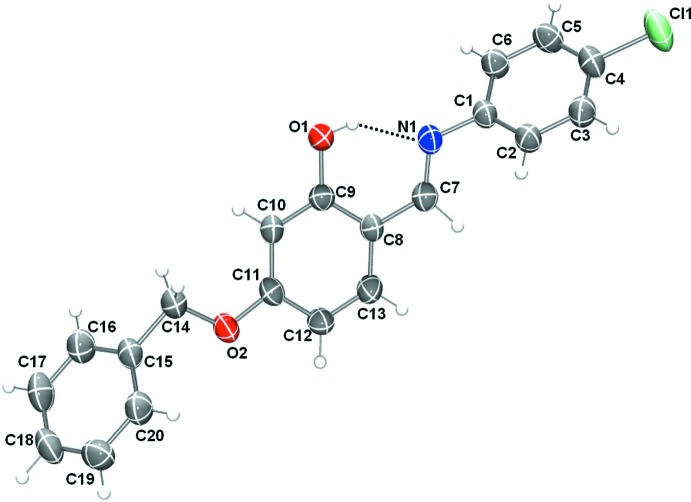
View of the mol­ecular structure of compound (I), with the atom labelling. Displacement ellipsoids are drawn at the 50% probability level. The intra­molecular O—H⋯N hydrogen bond (see Table 1 ▸) is shown as a dashed line.

The mol­ecular structures of compounds (I) and (II), illus­trated in Figs. 1 ▸ and 2 ▸, respectively, may be influenced by intra­molecular hydrogen bonds; O—H⋯N in (I) and N^+^—H⋯O^−^ in (II) (see Tables 1 ▸ and 2 ▸). These hydrogen bonds form *S*(6) ring motifs as shown in Figs. 1 ▸ and 2 ▸. In compound (II), the N atom is protonated (see *Section 6, Refinement*) and the C1—O13 (C—O^−^) bond length is 1.281 (2) Å, compared to the C9—O1 (C—OH) bond length of 1.343 (3) Å in (I). The configuration of the C=N imine bond is *E* in both compounds and the C=N bond lengths are 1.286 (3) Å for C7=N1 in (I) and 1.297 (3) Å for C11=N1 in (II). Neither mol­ecule is planar: in (I), the central benzene ring (C8–C13) is inclined to the two outer benzene rings (C1–C6 and C15–C20) by 53.52 (11) and 49.91 (12)°, respectively, while in (II) the central benzene ring (C12–C17) makes dihedral angles of 89.59 (9) and 72.27 (7)°, respectively, with outer benzene ring (C19–C24) and the mean plane of the indole ring system (N2/C1–C8; r.m.s. deviation = 0.011 Å).

## Supra­molecular features   

**Figure 3:**
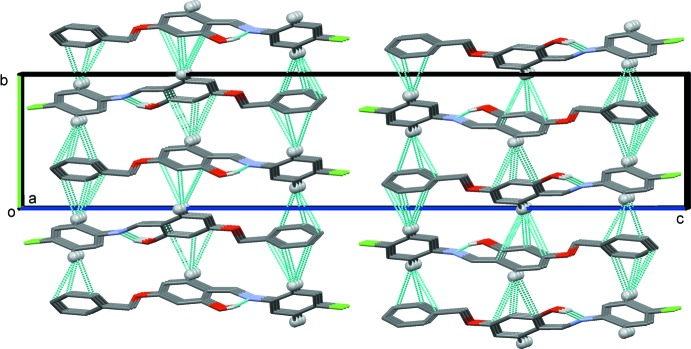
A view along the *a* axis of the crystal packing of compound (I). The intra­molecular O—H⋯N hydrogen bond and the inter­molecular C—H⋯π inter­actions are represented by dashed lines (see Table 1 ▸), and only the H atoms (grey balls) involved these inter­actions have been included.

**Figure 4:**
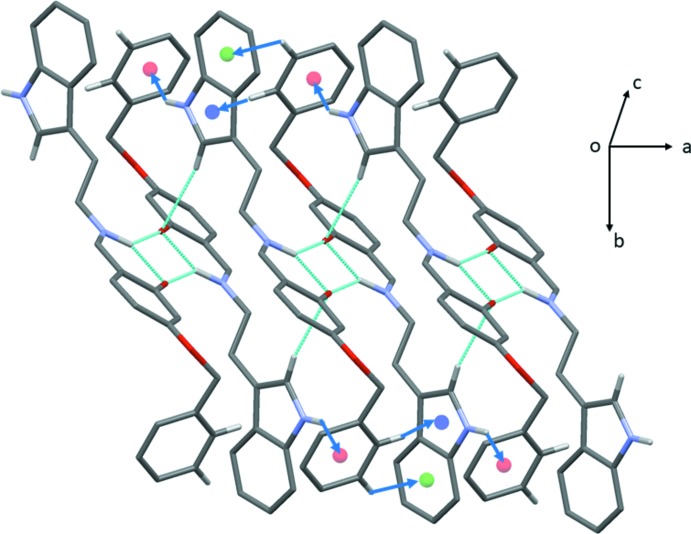
A view of the hydrogen bonds (dashed lines) and C—H⋯π and weak N—H⋯π inter­actions (blue arrows) in the crystal structure of compound (II); centroid *Cg*1 is blue, centroid *Cg*2 is green and centroid *Cg*4 is red (see Table 2 ▸). Only the H atoms involved in these inter­actions have been included.

**Figure 5:**
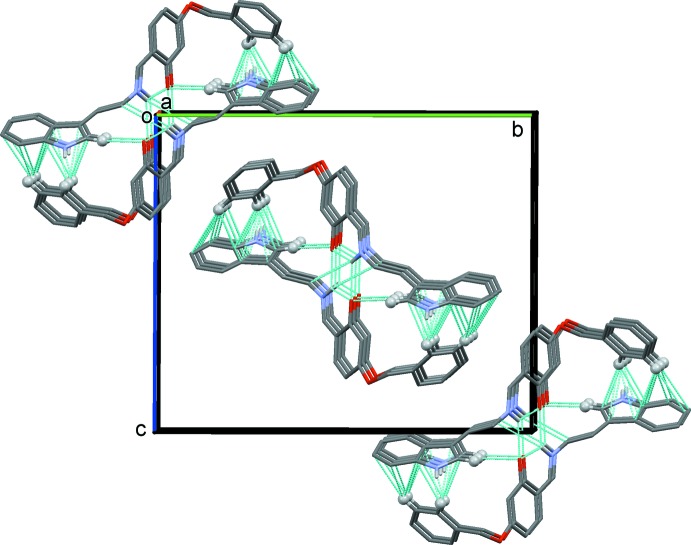
A view along the *a* axis of the crystal packing of compound (II). The hydrogen bonds and C—H⋯π inter­actions are shown as dashed lines (see Table 2 ▸) and only the H atoms involved in these inter­actions have been included.

In the crystal structures of both compounds C—H⋯π inter­actions predominate; see Table 1 ▸ for details concerning compound (I), and Table 2 ▸ for details concerning compound (II). In the crystal of (I), mol­ecules are linked by C—H⋯π inter­actions, forming slabs lying parallel to (001), as illustrated in Fig. 3 ▸. In the crystal of (II), mol­ecules are linked by pairs of N—H⋯O hydrogen bonds, forming inversion dimers. The dimers are linked by C—H⋯O hydrogen bonds and C—H⋯π inter­actions, and a weak N—H⋯π inter­action, forming columns propagating along the *a*-axis direction. The different hydrogen bonds and *X*—H⋯π (*X* = C, N) inter­actions are illustrated in Fig. 4 ▸, and the overall crystal packing is illus­trated in Fig. 5 ▸. There are no other significant inter­molecular contacts present in either crystal structure.

## Database survey   

The structures of Schiff bases derived from hydroxyaryl aldehydes have recently been the subject of a general survey, in which a number of structural errors, often involving misplaced H atoms, were pointed out (Blagus *et al.*, 2010 ▸). A search of the Cambridge Structural Database (Version 5.38, update May 2017; Groom *et al.*, 2016 ▸) for Schiff bases substituted by a phenol group gave over 900 hits. Of these only three compounds with a benzyl­oxyphenol group resemble the title compounds. They include, (*Z*)-3-benz­yloxy-6-[(2-hy­droxy­phenyl­amino)­methyl­ene]cyclo­hexa-2,4-dienone (KOS­CUS; Ghichi *et al.*, 2014*a* ▸), (*E*)-5-benz­yloxy-2-[(4-nitro­phenyl)carbonoimido­yl]phenol (RUTQOO; Ghichi *et al.*, 2015 ▸) and 5-benz­yloxy-2-{[(2-hy­droxy-5-methylphen­yl)iminio]methyl}phenolate (WOJBEE; Ghichi *et al.*, 2014*b* ▸). In RUTQOO there is an intra­molecular O—H⋯O hydrogen bond, as in compound (I). In KOSCUS and WOJBEE there are intra­molecular charge-assisted N^+^-H⋯O^−^ hydrogen bonds, as observed for compound (II).

## Anti­oxidant activity   

The anti­oxidant activity profile of the synthesized compound (I) was determined by utilizing the copper(II)–neocuprine (Cu^II^–Nc) (CUPRAC) method (Apak *et al.*, 2004 ▸). The CUPRAC method (cupric ion reducing anti­oxidant capacity) is based on the follow-up of the decrease in the increased absorbance of the neocuproene (Nc), copper (Cu^+2^)Nc_2_–Cu^+2^ complex. Indeed, in the presence of an anti­oxidant agent, the copper–neocuproene complex is reduced and this reaction is qu­anti­fied spectrophotometrically at a wavelength of 450 nm.

The current results indicate that Schiff base compound (I) has a low cupric ion reducing anti­oxidant capacity, because the absorbance in the CUPRAC assay is large (A_0.50_ > 100) for a 4 mg dosage (see Table 3 ▸). The current results indicate that the Schiff base compound (II), has a low free-radical scavenging activity (Blois, 1958 ▸), because the percentage inhibition in the DPPH assay is large (IC_50_ > 100) for a 1 mg dosage, by comparison with buthylated toluene (BHT) IC_50_ = 22.32 ±1.19, used as a positive control (see Table 3 ▸).

**Note:** Compound (I): the activity is cupric ion reducing anti­oxidant capacity (CUPRAC) with the BHT (positive control). Compound (II): the BHT positive control or standard reference is different for each anti­oxidant activity test (percentage inhibition).

## Synthesis and crystallization   

**Compound (I):**

2-Amino-4-chloro­phenol (1 equiv.) and 4-benz­yloxy-2-hy­droxy­benzaldehyde (1 equiv.) in ethanol (15 ml) were refluxed for 1 h. On completion of the reaction (monitored by thin layer chromatography), the solvent was evaporated *in vacuo*. The residue was recrystallized from methanol, yielding green block-like crystals of (I) on slow evaporation of the solvent. The purity of the compound was characterized by its NMR spectrum (250 MHz, CDCl_3_). In the ^1^H NMR spectrum, the azomethine proton appears in the 8.5–8.6 p.p.m. range, while the imine bond is characterized in the ^13^C MNR spectrum with the imine C signal in the 158–162 p.p.m. range. ^1^H NMR: δ 6.5–7.6 (*m*, 12H; *H-ar*), 13.8–14.0 (*s*, 1H; *OH*). ^13^C NMR: 70.22, 127.6, 128.8, 129.5 133.8, 136.2, 147.1.

**Compound (II):**

2-(2,3-Di­hydro-1*H*-indol-3-yl)ethan-1-amine (1 equiv.) and 4-benz­yloxy-2-hy­droxy­benzaldehyde (1 equiv.) in methanol (15 ml) were refluxed for 1 h. On completion of the reaction (monitored by thin layer chromatography), the solvent was evaporated *in vacuo* and the residue recrystallized from methanol, yielding orange block-like crystals of (II) on slow evaporation of the solvent. In the ^1^H NMR spectrum, the azomethine proton appears in the 8.5–8.6 p.p.m. range, while the imine bond is characterized in the ^13^C NMR spectrum with the imine C signal in the 163.3–168.4 p.p.m. range. ^1^H NMR: δ 6.5–7.7 (*m*, 14H; *H-ar*), 13.8–14.0 (*s*, 1H; *OH*). ^13^C NMR: 56.9, 128.2, 128.7, 132.9, 136.4, 163.3.

## Refinement   

Crystal data, data collection and structure refinement details are summarized in Table 4 ▸. In compound (I), the hydroxyl H atom was located in a difference-Fourier map and initially freely refined. In the final cycles of refinement it was positioned geometrically (O—H = 0.82 Å) and refined with *U*_iso_(H)= 1.5*U*_eq_(O). In compound (II), an H atom was located in a difference-Fourier map close to atom N1 of the C11=N1 bond, and was freely refined, as was the indole NH H atom. For both compounds, the C-bound H atoms were positioned geometrically (C—H = 0.93–0.97Å) and refined as riding with *U*_iso_(H) = 1.2*U*_eq_(C).

## Supplementary Material

Crystal structure: contains datablock(s) global, I, II. DOI: 10.1107/S2056989018003687/su5426sup1.cif

Supporting information file. DOI: 10.1107/S2056989018003687/su5426Isup2.cml

CCDC references: 1827172, 1827171

Additional supporting information:  crystallographic information; 3D view; checkCIF report

## Figures and Tables

**Figure 2:**
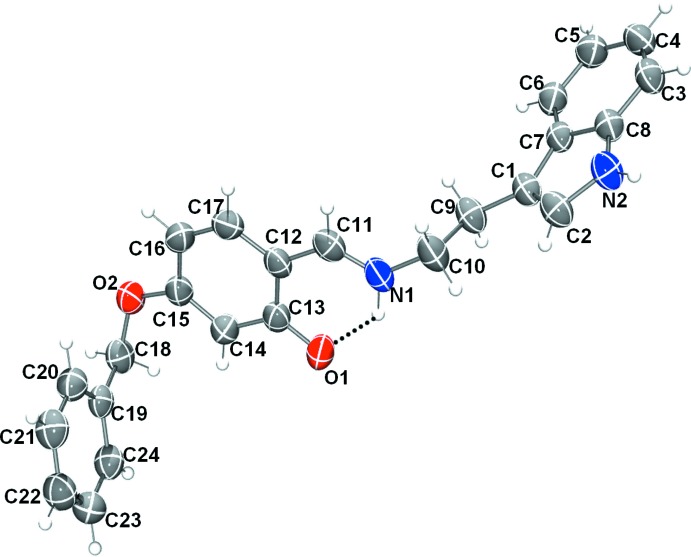
View of the mol­ecular structure of compound (II), with the atom labelling. Displacement ellipsoids are drawn at the 50% probability level. The intra­molecular charge-assisted N^+^—H⋯O^−^ hydrogen bond (see Table 2 ▸) is shown as a dashed line.

**Table 1:** Hydrogen-bond geometry (Å, °) for (I) *Cg*2 and *Cg*3 are the centroids of rings C8–C13 and C15–C20, respectively.

*D*—H⋯*A*	*D*—H	H⋯*A*	*D*⋯*A*	*D*—H⋯*A*
O1—H1*O*⋯N1	0.82	1.89	2.616 (3)	147
C3—H3⋯*Cg*3^i^	0.93	2.85	3.593 (3)	138
C6—H6⋯*Cg*3^ii^	0.93	2.82	3.520 (3)	133
C13—H13⋯*Cg*2^iii^	0.93	2.79	3.419 (3)	126

Symmetry codes: (i) 
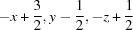
; (ii) 
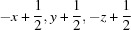
; (iii) 
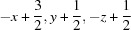
.

**Table 2:** Hydrogen-bond geometry (Å, °) for (II) *Cg*1, *Cg*2 and *Cg*4 are the centroids of rings N2/C1/C2/C7/C8, C3–C8 and C19–C24, respectively.

*D*—H⋯*A*	*D*—H	H⋯*A*	*D*⋯*A*	*D*—H⋯*A*
N1—H1*N*⋯O1	1.07 (3)	1.81 (3)	2.657 (2)	133 (2)
N1—H1*N*⋯O1^i^	1.07 (3)	2.19 (3)	3.004 (2)	131 (2)
C2—H2⋯O1^ii^	0.93	2.55	3.467 (2)	167
C23—H23⋯*Cg*2^i^	0.93	2.95	3.716 (2)	141
C24—H24⋯*Cg*1^i^	0.93	2.70	3.465 (3)	140
N2—H2*N*⋯*Cg*4^ii^	0.85 (2)	3.03 (2)	3.75 (3)	145 (2)

Symmetry codes: (i) 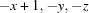; (ii) 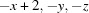.

**Table 3:** Cupric ion reducing anti­oxidant capacity of compound (I)

	Absorbances							
	12.5 µg	25 µg	50 µg	100 µg	200 µg	400 µg	800 µg	A0.50 (μg/ml)
Compound (I)	0.18±0.00	0.23±0.01	0.31±0.01	0.47±0.01	0.67±0.07	1.14±0.14	2.38±0.25	>100
BHT	1.41±0.03	2.22±0.05	2.42±0.02	2.50±0.01	2.56±0.05	2.86±0.07	3.38±0.13	8.97±3.94

**Table 4:** Experimental details

	(I)	(II)
Crystal data		
Chemical formula	C_20_H_16_ClNO_2_	C_24_H_22_N_2_O_2_
*M*_r_	337.79	370.43
Crystal system, space group	Monoclinic, *P*2_1_/*n*	Monoclinic, *P*2_1_/*c*
Temperature (K)	293	293
*a*, *b*, *c* (Å)	6.056 (2), 7.363 (3), 36.761 (12)	5.5265 (6), 20.1714 (19), 17.027 (2)
β (°)	91.30 (2)	97.216 (5)
*V* (Å^3^)	1638.6 (10)	1883.1 (4)
*Z*	4	4
Radiation type	Mo *K*α	Mo *K*α
μ (mm^−1^)	0.25	0.08
Crystal size (mm)	0.03 × 0.02 × 0.01	0.03 × 0.02 × 0.01

Data collection		
Diffractometer	Bruker APEXII CCD	Bruker APEXII CCD
No. of measured, independent and observed [*I* > 2σ(*I*)] reflections	13108, 3161, 2066	17491, 4255, 2304
*R*_int_	0.053	0.053
(sin θ/λ)_max_ (Å^−1^)	0.617	0.650

Refinement		
*R*[*F* ^2^ > 2σ(*F* ^2^)], *wR*(*F* ^2^), *S*	0.053, 0.153, 1.05	0.047, 0.124, 1.00
No. of reflections	3161	4255
No. of parameters	221	265
H-atom treatment	H atoms treated by a mixture of independent and constrained refinement	H atoms treated by a mixture of independent and constrained refinement
Δρ_max_, Δρ_min_ (e Å^−3^)	0.35, −0.22	0.14, −0.16

Computer programs: *APEX2* and *SAINT* (Bruker, 2012 ▸), *SHELXS97* (Sheldrick, 2008 ▸), *SHELXL2017* and, *SHELXL2014* (Sheldrick, 2015 ▸), *SHELXTL* (Sheldrick, 2008 ▸), *Mercury* (Macrae *et al.*, 2008 ▸) and *PLATON* (Spek, 2009 ▸).

## supplementary crystallographic information

### (E)-5-Benzyloxy-2-{[(4-chlorophenyl)imino]methyl}phenol (I) Crystal data

**Table:**

C_20_H_16_ClNO_2_	*F*(000) = 704
*M**_r_* = 337.79	*D*_x_ = 1.369 Mg m^−^^3^
Monoclinic, *P*2_1_/*n*	Mo *K*α radiation, λ = 0.71073 Å
*a* = 6.056 (2) Å	Cell parameters from 2596 reflections
*b* = 7.363 (3) Å	θ = 3.0–22.7°
*c* = 36.761 (12) Å	µ = 0.25 mm^−^^1^
β = 91.30 (2)°	*T* = 293 K
*V* = 1638.6 (10) Å^3^	Block, green
*Z* = 4	0.03 × 0.02 × 0.01 mm

### (E)-5-Benzyloxy-2-{[(4-chlorophenyl)imino]methyl}phenol (I) Data collection

**Table:**

Bruker APEXII CCD diffractometer	*R*_int_ = 0.053
Detector resolution: 18.4 pixels mm^-1^	θ_max_ = 26.0°, θ_min_ = 2.2°
φ and ω scans	*h* = −7→6
13108 measured reflections	*k* = −9→9
3161 independent reflections	*l* = −45→45
2066 reflections with *I* > 2σ(*I*)	

### (E)-5-Benzyloxy-2-{[(4-chlorophenyl)imino]methyl}phenol (I) Refinement

**Table:**

Refinement on *F*^2^	Primary atom site location: structure-invariant direct methods
Least-squares matrix: full	Secondary atom site location: difference Fourier map
*R*[*F*^2^ > 2σ(*F*^2^)] = 0.053	Hydrogen site location: mixed
*wR*(*F*^2^) = 0.153	H atoms treated by a mixture of independent and constrained refinement
*S* = 1.05	*w* = 1/[σ^2^(*F*_o_^2^) + (0.0611*P*)^2^ + 0.6069*P*] where *P* = (*F*_o_^2^ + 2*F*_c_^2^)/3
3161 reflections	(Δ/σ)_max_ < 0.001
221 parameters	Δρ_max_ = 0.35 e Å^−^^3^
0 restraints	Δρ_min_ = −0.22 e Å^−^^3^

### (E)-5-Benzyloxy-2-{[(4-chlorophenyl)imino]methyl}phenol (I) Special details

**Table:**

Geometry. Bond distances, angles etc. have been calculated using the rounded fractional coordinates. All su's are estimated from the variances of the (full) variance-covariance matrix. The cell esds are taken into account in the estimation of distances, angles and torsion angles

### (E)-5-Benzyloxy-2-{[(4-chlorophenyl)imino]methyl}phenol (I) Fractional atomic coordinates and isotropic or equivalent isotropic displacement parameters (Å^2^)

**Table:**

	*x*	*y*	*z*	*U*_iso_*/*U*_eq_	
Cl1	1.14818 (15)	0.25490 (13)	0.48315 (2)	0.0818 (3)	
O1	0.2926 (3)	0.2500 (3)	0.30360 (5)	0.0557 (7)	
O2	0.3778 (3)	0.3501 (2)	0.17647 (4)	0.0492 (6)	
N1	0.6324 (3)	0.3275 (3)	0.34598 (5)	0.0424 (7)	
C1	0.7583 (4)	0.3110 (3)	0.37880 (6)	0.0397 (8)	
C2	0.9632 (4)	0.2267 (3)	0.37907 (7)	0.0436 (8)	
C3	1.0823 (4)	0.2078 (3)	0.41147 (7)	0.0480 (8)	
C4	0.9972 (5)	0.2739 (4)	0.44320 (7)	0.0504 (9)	
C5	0.7914 (4)	0.3564 (4)	0.44332 (7)	0.0514 (9)	
C6	0.6721 (4)	0.3727 (3)	0.41101 (6)	0.0464 (8)	
C7	0.7282 (4)	0.3864 (3)	0.31744 (6)	0.0411 (8)	
C8	0.6226 (4)	0.3874 (3)	0.28193 (6)	0.0367 (7)	
C9	0.4121 (4)	0.3121 (3)	0.27590 (6)	0.0389 (7)	
C10	0.3232 (4)	0.2973 (3)	0.24089 (6)	0.0402 (8)	
C11	0.4436 (4)	0.3603 (3)	0.21186 (6)	0.0393 (8)	
C12	0.6490 (4)	0.4408 (3)	0.21739 (6)	0.0435 (8)	
C13	0.7361 (4)	0.4520 (3)	0.25213 (6)	0.0445 (8)	
C14	0.1774 (4)	0.2582 (4)	0.16730 (7)	0.0530 (9)	
C15	0.1428 (4)	0.2742 (3)	0.12690 (6)	0.0440 (8)	
C16	−0.0498 (4)	0.3482 (4)	0.11281 (7)	0.0538 (9)	
C17	−0.0834 (5)	0.3639 (4)	0.07556 (8)	0.0638 (11)	
C18	0.0762 (5)	0.3076 (4)	0.05245 (7)	0.0613 (10)	
C19	0.2689 (5)	0.2336 (4)	0.06624 (8)	0.0597 (10)	
C20	0.3032 (4)	0.2173 (4)	0.10328 (7)	0.0517 (9)	
H1O	0.36110	0.26625	0.32283	0.0830*	
H2	1.02053	0.18292	0.35751	0.0520*	
H3	1.21931	0.15070	0.41176	0.0580*	
H5	0.73435	0.40013	0.46490	0.0620*	
H6	0.53266	0.42571	0.41093	0.0560*	
H7	0.889 (4)	0.431 (3)	0.3166 (6)	0.055 (7)*	
H10	0.18449	0.24578	0.23697	0.0480*	
H12	0.72641	0.48643	0.19784	0.0520*	
H13	0.87466	0.50422	0.25581	0.0530*	
H14A	0.05497	0.31303	0.17984	0.0640*	
H14B	0.18748	0.13143	0.17434	0.0640*	
H16	−0.15821	0.38799	0.12846	0.0650*	
H17	−0.21464	0.41268	0.06628	0.0760*	
H18	0.05454	0.31939	0.02744	0.0730*	
H19	0.37694	0.19433	0.05046	0.0720*	
H20	0.43446	0.16793	0.11241	0.0620*	

### (E)-5-Benzyloxy-2-{[(4-chlorophenyl)imino]methyl}phenol (I) Atomic displacement parameters (Å^2^)

**Table:**

	*U*^11^	*U*^22^	*U*^33^	*U*^12^	*U*^13^	*U*^23^
Cl1	0.0886 (6)	0.1109 (7)	0.0449 (5)	0.0212 (5)	−0.0232 (4)	−0.0042 (4)
O1	0.0456 (10)	0.0844 (14)	0.0369 (10)	−0.0174 (9)	−0.0005 (7)	0.0024 (9)
O2	0.0542 (10)	0.0571 (11)	0.0359 (10)	−0.0125 (9)	−0.0058 (8)	0.0028 (8)
N1	0.0414 (11)	0.0466 (12)	0.0390 (12)	−0.0013 (9)	−0.0041 (9)	−0.0028 (9)
C1	0.0422 (13)	0.0400 (13)	0.0367 (13)	−0.0024 (10)	−0.0045 (10)	0.0001 (10)
C2	0.0442 (13)	0.0491 (15)	0.0374 (13)	0.0005 (11)	−0.0001 (10)	−0.0041 (11)
C3	0.0438 (13)	0.0526 (16)	0.0474 (15)	0.0030 (12)	−0.0018 (11)	−0.0024 (12)
C4	0.0576 (16)	0.0536 (16)	0.0396 (14)	0.0001 (13)	−0.0095 (12)	0.0003 (12)
C5	0.0613 (16)	0.0562 (17)	0.0368 (14)	0.0057 (13)	0.0048 (12)	−0.0068 (12)
C6	0.0484 (14)	0.0505 (15)	0.0402 (14)	0.0064 (12)	0.0016 (11)	−0.0051 (11)
C7	0.0430 (13)	0.0366 (13)	0.0437 (14)	−0.0017 (11)	−0.0018 (11)	−0.0021 (11)
C8	0.0380 (12)	0.0356 (12)	0.0364 (13)	0.0008 (10)	−0.0034 (10)	−0.0024 (10)
C9	0.0382 (12)	0.0406 (13)	0.0379 (13)	0.0004 (10)	0.0032 (10)	0.0003 (10)
C10	0.0373 (12)	0.0433 (14)	0.0398 (13)	−0.0020 (10)	−0.0051 (10)	−0.0016 (11)
C11	0.0465 (13)	0.0358 (13)	0.0354 (13)	0.0021 (11)	−0.0036 (10)	0.0010 (10)
C12	0.0463 (13)	0.0440 (14)	0.0403 (14)	−0.0076 (11)	0.0013 (11)	0.0038 (11)
C13	0.0423 (13)	0.0447 (14)	0.0464 (15)	−0.0094 (11)	−0.0026 (11)	0.0024 (11)
C14	0.0505 (15)	0.0669 (18)	0.0413 (14)	−0.0079 (13)	−0.0051 (11)	0.0009 (12)
C15	0.0474 (14)	0.0449 (14)	0.0392 (14)	−0.0025 (11)	−0.0070 (11)	0.0000 (11)
C16	0.0528 (15)	0.0557 (17)	0.0527 (17)	0.0055 (13)	−0.0036 (12)	−0.0050 (13)
C17	0.0638 (18)	0.0645 (19)	0.062 (2)	0.0034 (15)	−0.0239 (15)	0.0037 (15)
C18	0.077 (2)	0.0672 (19)	0.0389 (15)	−0.0091 (16)	−0.0153 (14)	0.0024 (14)
C19	0.0669 (18)	0.0667 (19)	0.0459 (16)	−0.0072 (15)	0.0083 (13)	−0.0092 (14)
C20	0.0486 (14)	0.0564 (17)	0.0497 (16)	0.0035 (12)	−0.0049 (12)	0.0003 (13)

### (E)-5-Benzyloxy-2-{[(4-chlorophenyl)imino]methyl}phenol (I) Geometric parameters (Å, º)

**Table:**

Cl1—C4	1.718 (3)	C15—C20	1.383 (3)
O1—C9	1.343 (3)	C15—C16	1.378 (4)
O2—C11	1.354 (3)	C16—C17	1.385 (4)
O2—C14	1.423 (3)	C17—C18	1.366 (4)
N1—C1	1.418 (3)	C18—C19	1.374 (4)
N1—C7	1.286 (3)	C19—C20	1.378 (4)
O1—H1O	0.8200	C2—H2	0.9300
C1—C2	1.387 (3)	C3—H3	0.9300
C1—C6	1.382 (3)	C5—H5	0.9300
C2—C3	1.385 (4)	C6—H6	0.9300
C3—C4	1.375 (4)	C7—H7	1.03 (2)
C4—C5	1.387 (4)	C10—H10	0.9300
C5—C6	1.381 (3)	C12—H12	0.9300
C7—C8	1.441 (3)	C13—H13	0.9300
C8—C13	1.390 (3)	C14—H14A	0.9700
C8—C9	1.403 (3)	C14—H14B	0.9700
C9—C10	1.388 (3)	C16—H16	0.9300
C10—C11	1.386 (3)	C17—H17	0.9300
C11—C12	1.389 (3)	C18—H18	0.9300
C12—C13	1.373 (3)	C19—H19	0.9300
C14—C15	1.500 (3)	C20—H20	0.9300
			
C11—O2—C14	118.99 (18)	C18—C19—C20	120.5 (3)
C1—N1—C7	118.7 (2)	C15—C20—C19	120.1 (2)
C9—O1—H1O	109.00	C1—C2—H2	120.00
N1—C1—C2	120.5 (2)	C3—C2—H2	120.00
C2—C1—C6	119.8 (2)	C2—C3—H3	120.00
N1—C1—C6	119.7 (2)	C4—C3—H3	120.00
C1—C2—C3	120.0 (2)	C4—C5—H5	120.00
C2—C3—C4	119.7 (2)	C6—C5—H5	120.00
Cl1—C4—C3	119.6 (2)	C1—C6—H6	120.00
Cl1—C4—C5	119.6 (2)	C5—C6—H6	120.00
C3—C4—C5	120.8 (2)	N1—C7—H7	125.3 (12)
C4—C5—C6	119.2 (2)	C8—C7—H7	111.9 (12)
C1—C6—C5	120.5 (2)	C9—C10—H10	120.00
N1—C7—C8	122.8 (2)	C11—C10—H10	120.00
C7—C8—C13	119.9 (2)	C11—C12—H12	120.00
C9—C8—C13	118.3 (2)	C13—C12—H12	120.00
C7—C8—C9	121.6 (2)	C8—C13—H13	119.00
O1—C9—C10	118.2 (2)	C12—C13—H13	119.00
C8—C9—C10	120.6 (2)	O2—C14—H14A	110.00
O1—C9—C8	121.2 (2)	O2—C14—H14B	110.00
C9—C10—C11	119.2 (2)	C15—C14—H14A	110.00
O2—C11—C12	114.0 (2)	C15—C14—H14B	110.00
C10—C11—C12	121.0 (2)	H14A—C14—H14B	109.00
O2—C11—C10	124.9 (2)	C15—C16—H16	120.00
C11—C12—C13	119.1 (2)	C17—C16—H16	120.00
C8—C13—C12	121.7 (2)	C16—C17—H17	120.00
O2—C14—C15	107.2 (2)	C18—C17—H17	120.00
C14—C15—C16	120.1 (2)	C17—C18—H18	120.00
C16—C15—C20	119.0 (2)	C19—C18—H18	120.00
C14—C15—C20	120.9 (2)	C18—C19—H19	120.00
C15—C16—C17	120.6 (2)	C20—C19—H19	120.00
C16—C17—C18	120.0 (3)	C15—C20—H20	120.00
C17—C18—C19	119.9 (3)	C19—C20—H20	120.00
			
C14—O2—C11—C10	−4.3 (3)	C13—C8—C9—C10	2.1 (3)
C14—O2—C11—C12	175.4 (2)	C7—C8—C13—C12	174.2 (2)
C11—O2—C14—C15	178.00 (19)	C9—C8—C13—C12	−1.1 (3)
C7—N1—C1—C2	−47.9 (3)	O1—C9—C10—C11	−179.9 (2)
C7—N1—C1—C6	134.8 (2)	C8—C9—C10—C11	−0.9 (3)
C1—N1—C7—C8	172.3 (2)	C9—C10—C11—O2	178.4 (2)
N1—C1—C2—C3	−178.5 (2)	C9—C10—C11—C12	−1.3 (3)
C6—C1—C2—C3	−1.2 (3)	O2—C11—C12—C13	−177.5 (2)
N1—C1—C6—C5	179.4 (2)	C10—C11—C12—C13	2.2 (3)
C2—C1—C6—C5	2.1 (4)	C11—C12—C13—C8	−1.0 (3)
C1—C2—C3—C4	−0.4 (4)	O2—C14—C15—C16	−124.0 (2)
C2—C3—C4—Cl1	−178.50 (19)	O2—C14—C15—C20	55.3 (3)
C2—C3—C4—C5	1.2 (4)	C14—C15—C16—C17	179.9 (3)
Cl1—C4—C5—C6	179.4 (2)	C20—C15—C16—C17	0.6 (4)
C3—C4—C5—C6	−0.4 (4)	C14—C15—C20—C19	−179.8 (3)
C4—C5—C6—C1	−1.3 (4)	C16—C15—C20—C19	−0.5 (4)
N1—C7—C8—C9	−4.4 (4)	C15—C16—C17—C18	−0.7 (4)
N1—C7—C8—C13	−179.6 (2)	C16—C17—C18—C19	0.7 (4)
C7—C8—C9—O1	5.8 (3)	C17—C18—C19—C20	−0.6 (5)
C7—C8—C9—C10	−173.2 (2)	C18—C19—C20—C15	0.5 (4)
C13—C8—C9—O1	−178.9 (2)		

### (E)-5-Benzyloxy-2-{[(4-chlorophenyl)imino]methyl}phenol (I) Hydrogen-bond geometry (Å, º)

Cg2 and Cg3 are the centroids of rings C8–C13 and C15–C20, respectively.

**Table:**

*D*—H···*A*	*D*—H	H···*A*	*D*···*A*	*D*—H···*A*
O1—H1*O*···N1	0.82	1.89	2.616 (3)	147
C3—H3···*Cg*3^i^	0.93	2.85	3.593 (3)	138
C6—H6···*Cg*3^ii^	0.93	2.82	3.520 (3)	133
C13—H13···*Cg*2^iii^	0.93	2.79	3.419 (3)	126

Symmetry codes: (i) −*x*+3/2, *y*−1/2, −*z*+1/2; (ii) −*x*+1/2, *y*+1/2, −*z*+1/2; (iii) −*x*+3/2, *y*+1/2, −*z*+1/2.

### (E)-5-Benzyloxy-2-({[2-(1H-indol-3-yl)ethyl]iminiumyl}methyl)phenolate (II) Crystal data

**Table:**

C_24_H_22_N_2_O_2_	*F*(000) = 784
*M**_r_* = 370.43	*D*_x_ = 1.307 Mg m^−^^3^
Monoclinic, *P*2_1_/*c*	Mo *K*α radiation, λ = 0.71073 Å
*a* = 5.5265 (6) Å	Cell parameters from 2857 reflections
*b* = 20.1714 (19) Å	θ = 3.2–23.1°
*c* = 17.027 (2) Å	µ = 0.08 mm^−^^1^
β = 97.216 (5)°	*T* = 293 K
*V* = 1883.1 (4) Å^3^	Block, orange
*Z* = 4	0.03 × 0.02 × 0.01 mm

### (E)-5-Benzyloxy-2-({[2-(1H-indol-3-yl)ethyl]iminiumyl}methyl)phenolate (II) Data collection

**Table:**

Bruker APEXII CCD diffractometer	*R*_int_ = 0.053
Detector resolution: 18.4 pixels mm^-1^	θ_max_ = 27.5°, θ_min_ = 3.7°
φ and ω scans	*h* = −6→7
17491 measured reflections	*k* = −20→26
4255 independent reflections	*l* = −22→21
2304 reflections with *I* > 2σ(*I*)	

### (E)-5-Benzyloxy-2-({[2-(1H-indol-3-yl)ethyl]iminiumyl}methyl)phenolate (II) Refinement

**Table:**

Refinement on *F*^2^	Primary atom site location: structure-invariant direct methods
Least-squares matrix: full	Secondary atom site location: difference Fourier map
*R*[*F*^2^ > 2σ(*F*^2^)] = 0.047	Hydrogen site location: mixed
*wR*(*F*^2^) = 0.124	H atoms treated by a mixture of independent and constrained refinement
*S* = 1.00	*w* = 1/[σ^2^(*F*_o_^2^) + (0.054*P*)^2^] where *P* = (*F*_o_^2^ + 2*F*_c_^2^)/3
4255 reflections	(Δ/σ)_max_ < 0.001
265 parameters	Δρ_max_ = 0.14 e Å^−^^3^
0 restraints	Δρ_min_ = −0.16 e Å^−^^3^

### (E)-5-Benzyloxy-2-({[2-(1H-indol-3-yl)ethyl]iminiumyl}methyl)phenolate (II) Special details

**Table:**

Geometry. Bond distances, angles etc. have been calculated using the rounded fractional coordinates. All su's are estimated from the variances of the (full) variance-covariance matrix. The cell esds are taken into account in the estimation of distances, angles and torsion angles

### (E)-5-Benzyloxy-2-({[2-(1H-indol-3-yl)ethyl]iminiumyl}methyl)phenolate (II) Fractional atomic coordinates and isotropic or equivalent isotropic displacement parameters (Å^2^)

**Table:**

	*x*	*y*	*z*	*U*_iso_*/*U*_eq_	
O1	0.4706 (2)	−0.02601 (6)	0.07995 (7)	0.0650 (5)	
O2	0.2598 (2)	−0.07547 (6)	0.33695 (7)	0.0583 (4)	
N1	0.8488 (3)	0.05404 (7)	0.07254 (10)	0.0556 (6)	
N2	1.3886 (3)	0.23680 (8)	−0.09864 (10)	0.0562 (6)	
C1	1.0855 (3)	0.20232 (8)	−0.03230 (10)	0.0472 (6)	
C2	1.2742 (3)	0.18243 (9)	−0.07135 (11)	0.0541 (6)	
C3	1.3252 (3)	0.35979 (9)	−0.08998 (10)	0.0553 (6)	
C4	1.1802 (4)	0.40612 (10)	−0.06064 (11)	0.0599 (7)	
C5	0.9873 (4)	0.38760 (9)	−0.02008 (10)	0.0598 (7)	
C6	0.9357 (3)	0.32199 (9)	−0.00793 (10)	0.0529 (6)	
C7	1.0829 (3)	0.27313 (8)	−0.03608 (9)	0.0436 (5)	
C8	1.2754 (3)	0.29322 (9)	−0.07754 (10)	0.0456 (6)	
C9	0.9184 (3)	0.16023 (9)	0.00909 (12)	0.0554 (6)	
C10	1.0162 (3)	0.09284 (9)	0.03068 (12)	0.0614 (7)	
C11	0.8718 (4)	0.04992 (9)	0.14913 (13)	0.0550 (7)	
C12	0.7144 (3)	0.01537 (8)	0.19354 (10)	0.0479 (6)	
C13	0.5134 (3)	−0.02203 (8)	0.15551 (10)	0.0488 (6)	
C14	0.3631 (3)	−0.05484 (9)	0.20536 (10)	0.0514 (6)	
C15	0.4034 (3)	−0.04853 (8)	0.28556 (10)	0.0482 (6)	
C16	0.6007 (4)	−0.01132 (9)	0.32228 (11)	0.0553 (6)	
C17	0.7519 (4)	0.01853 (9)	0.27684 (11)	0.0557 (7)	
C18	0.0976 (3)	−0.12825 (9)	0.30843 (11)	0.0555 (6)	
C19	0.2329 (3)	−0.19171 (9)	0.29849 (10)	0.0468 (6)	
C20	0.4546 (3)	−0.20537 (10)	0.34351 (10)	0.0551 (7)	
C21	0.5697 (3)	−0.26495 (10)	0.33539 (12)	0.0633 (7)	
C22	0.4657 (4)	−0.31237 (10)	0.28371 (12)	0.0650 (7)	
C23	0.2467 (4)	−0.29912 (10)	0.23859 (12)	0.0639 (8)	
C24	0.1331 (3)	−0.23922 (10)	0.24611 (11)	0.0562 (7)	
H2N	1.506 (4)	0.2359 (10)	−0.1260 (11)	0.069 (6)*	
H2	1.31882	0.13859	−0.07844	0.0650*	
H1N	0.692 (5)	0.0289 (12)	0.0442 (16)	0.124 (9)*	
H3	1.45310	0.37232	−0.11740	0.0660*	
H4	1.21088	0.45089	−0.06789	0.0720*	
H5	0.89146	0.42023	−0.00084	0.0720*	
H6	0.80493	0.31023	0.01861	0.0630*	
H9A	0.88553	0.18281	0.05692	0.0670*	
H9B	0.76469	0.15557	−0.02483	0.0670*	
H10A	1.04499	0.06948	−0.01707	0.0740*	
H10B	1.17118	0.09713	0.06398	0.0740*	
H11	1.010 (3)	0.0728 (8)	0.1783 (9)	0.050 (5)*	
H14	0.23468	−0.08114	0.18269	0.0620*	
H16	0.62682	−0.00730	0.37710	0.0660*	
H17	0.88535	0.04199	0.30136	0.0670*	
H18A	0.00844	−0.11556	0.25795	0.0670*	
H18B	−0.01974	−0.13537	0.34543	0.0670*	
H20	0.52553	−0.17406	0.37934	0.0660*	
H21	0.71946	−0.27329	0.36514	0.0760*	
H22	0.54258	−0.35297	0.27933	0.0780*	
H23	0.17556	−0.33064	0.20306	0.0770*	
H24	−0.01436	−0.23062	0.21512	0.0670*	

### (E)-5-Benzyloxy-2-({[2-(1H-indol-3-yl)ethyl]iminiumyl}methyl)phenolate (II) Atomic displacement parameters (Å^2^)

**Table:**

	*U*^11^	*U*^22^	*U*^33^	*U*^12^	*U*^13^	*U*^23^
O1	0.0792 (9)	0.0719 (9)	0.0439 (8)	−0.0150 (7)	0.0081 (7)	0.0055 (6)
O2	0.0718 (8)	0.0555 (8)	0.0511 (7)	−0.0016 (7)	0.0216 (6)	−0.0004 (6)
N1	0.0630 (10)	0.0418 (9)	0.0651 (11)	−0.0024 (8)	0.0200 (8)	0.0074 (8)
N2	0.0456 (9)	0.0539 (10)	0.0714 (10)	0.0000 (8)	0.0165 (8)	0.0095 (8)
C1	0.0403 (9)	0.0470 (11)	0.0526 (10)	−0.0021 (8)	−0.0007 (8)	0.0092 (8)
C2	0.0458 (10)	0.0463 (11)	0.0696 (12)	0.0011 (9)	0.0047 (9)	0.0089 (9)
C3	0.0506 (10)	0.0559 (12)	0.0593 (11)	−0.0095 (9)	0.0069 (9)	0.0097 (9)
C4	0.0704 (12)	0.0451 (11)	0.0626 (12)	−0.0082 (10)	0.0016 (10)	0.0040 (9)
C5	0.0717 (13)	0.0520 (12)	0.0561 (11)	0.0035 (10)	0.0093 (10)	−0.0014 (9)
C6	0.0526 (10)	0.0593 (12)	0.0466 (10)	−0.0031 (9)	0.0058 (8)	0.0031 (9)
C7	0.0415 (9)	0.0470 (10)	0.0407 (9)	−0.0025 (8)	−0.0010 (7)	0.0053 (8)
C8	0.0394 (9)	0.0485 (11)	0.0479 (10)	−0.0015 (8)	0.0017 (8)	0.0055 (8)
C9	0.0445 (10)	0.0519 (11)	0.0695 (12)	−0.0050 (8)	0.0058 (9)	0.0122 (9)
C10	0.0643 (12)	0.0468 (11)	0.0775 (13)	0.0016 (10)	0.0257 (10)	0.0093 (10)
C11	0.0576 (12)	0.0395 (11)	0.0685 (13)	0.0035 (9)	0.0105 (10)	0.0014 (9)
C12	0.0552 (10)	0.0349 (9)	0.0546 (11)	0.0021 (8)	0.0107 (9)	0.0049 (8)
C13	0.0584 (11)	0.0403 (10)	0.0485 (11)	0.0053 (8)	0.0101 (9)	0.0025 (8)
C14	0.0555 (10)	0.0516 (11)	0.0469 (11)	−0.0051 (9)	0.0054 (8)	0.0002 (8)
C15	0.0571 (11)	0.0409 (10)	0.0486 (10)	0.0050 (9)	0.0147 (9)	0.0008 (8)
C16	0.0718 (12)	0.0478 (11)	0.0463 (10)	0.0030 (10)	0.0075 (10)	−0.0037 (8)
C17	0.0617 (11)	0.0449 (11)	0.0588 (12)	−0.0043 (9)	0.0013 (10)	−0.0025 (9)
C18	0.0518 (10)	0.0597 (12)	0.0575 (11)	−0.0010 (9)	0.0162 (9)	0.0061 (9)
C19	0.0458 (9)	0.0527 (11)	0.0434 (10)	−0.0029 (8)	0.0111 (8)	0.0072 (8)
C20	0.0528 (11)	0.0596 (12)	0.0524 (11)	−0.0043 (9)	0.0047 (9)	0.0010 (9)
C21	0.0525 (11)	0.0713 (14)	0.0644 (12)	0.0054 (11)	0.0003 (10)	0.0095 (11)
C22	0.0689 (13)	0.0560 (12)	0.0708 (13)	0.0090 (11)	0.0114 (11)	0.0046 (11)
C23	0.0703 (13)	0.0573 (13)	0.0636 (13)	−0.0063 (11)	0.0060 (11)	−0.0024 (10)
C24	0.0507 (10)	0.0630 (13)	0.0537 (11)	−0.0023 (10)	0.0023 (9)	0.0067 (9)

### (E)-5-Benzyloxy-2-({[2-(1H-indol-3-yl)ethyl]iminiumyl}methyl)phenolate (II) Geometric parameters (Å, º)

**Table:**

O1—C13	1.281 (2)	C19—C24	1.376 (3)
O2—C15	1.366 (2)	C19—C20	1.389 (2)
O2—C18	1.437 (2)	C20—C21	1.375 (3)
N1—C10	1.464 (2)	C21—C22	1.376 (3)
N1—C11	1.297 (3)	C22—C23	1.376 (3)
N2—C2	1.376 (2)	C23—C24	1.375 (3)
N2—C8	1.368 (2)	C2—H2	0.9300
C1—C2	1.366 (2)	C3—H3	0.9300
C1—C7	1.430 (2)	C4—H4	0.9300
C1—C9	1.495 (2)	C5—H5	0.9300
N1—H1N	1.07 (3)	C6—H6	0.9300
N2—H2N	0.85 (2)	C9—H9A	0.9700
C3—C8	1.392 (3)	C9—H9B	0.9700
C3—C4	1.366 (3)	C10—H10A	0.9700
C4—C5	1.392 (3)	C10—H10B	0.9700
C5—C6	1.375 (3)	C11—H11	0.974 (16)
C6—C7	1.400 (2)	C14—H14	0.9300
C7—C8	1.408 (2)	C16—H16	0.9300
C9—C10	1.492 (3)	C17—H17	0.9300
C11—C12	1.407 (3)	C18—H18A	0.9700
C12—C17	1.409 (3)	C18—H18B	0.9700
C12—C13	1.429 (2)	C20—H20	0.9300
C13—C14	1.423 (2)	C21—H21	0.9300
C14—C15	1.362 (2)	C22—H22	0.9300
C15—C16	1.404 (3)	C23—H23	0.9300
C16—C17	1.350 (3)	C24—H24	0.9300
C18—C19	1.503 (3)		
			
C15—O2—C18	117.81 (13)	C19—C24—C23	121.45 (17)
C10—N1—C11	122.40 (17)	N2—C2—H2	125.00
C2—N2—C8	109.25 (15)	C1—C2—H2	125.00
C2—C1—C7	106.00 (15)	C4—C3—H3	121.00
C2—C1—C9	128.14 (16)	C8—C3—H3	121.00
C7—C1—C9	125.83 (15)	C3—C4—H4	119.00
C10—N1—H1N	124.2 (15)	C5—C4—H4	119.00
C11—N1—H1N	113.3 (15)	C4—C5—H5	119.00
N2—C2—C1	109.98 (16)	C6—C5—H5	119.00
C2—N2—H2N	125.9 (14)	C5—C6—H6	120.00
C8—N2—H2N	124.8 (14)	C7—C6—H6	120.00
C4—C3—C8	117.90 (16)	C1—C9—H9A	109.00
C3—C4—C5	121.26 (18)	C1—C9—H9B	109.00
C4—C5—C6	121.28 (18)	C10—C9—H9A	109.00
C5—C6—C7	119.05 (16)	C10—C9—H9B	109.00
C6—C7—C8	118.49 (15)	H9A—C9—H9B	108.00
C1—C7—C8	107.82 (15)	N1—C10—H10A	109.00
C1—C7—C6	133.69 (16)	N1—C10—H10B	109.00
N2—C8—C7	106.94 (15)	C9—C10—H10A	109.00
C3—C8—C7	122.01 (16)	C9—C10—H10B	109.00
N2—C8—C3	131.04 (16)	H10A—C10—H10B	108.00
C1—C9—C10	114.06 (14)	N1—C11—H11	117.0 (9)
N1—C10—C9	112.07 (14)	C12—C11—H11	117.3 (9)
N1—C11—C12	125.67 (19)	C13—C14—H14	119.00
C11—C12—C13	121.06 (16)	C15—C14—H14	119.00
C11—C12—C17	119.73 (17)	C15—C16—H16	120.00
C13—C12—C17	119.20 (16)	C17—C16—H16	120.00
O1—C13—C14	121.46 (15)	C12—C17—H17	119.00
C12—C13—C14	117.00 (15)	C16—C17—H17	119.00
O1—C13—C12	121.55 (15)	O2—C18—H18A	109.00
C13—C14—C15	121.36 (16)	O2—C18—H18B	109.00
C14—C15—C16	121.10 (16)	C19—C18—H18A	109.00
O2—C15—C14	124.77 (15)	C19—C18—H18B	109.00
O2—C15—C16	114.12 (15)	H18A—C18—H18B	108.00
C15—C16—C17	119.03 (17)	C19—C20—H20	120.00
C12—C17—C16	122.24 (18)	C21—C20—H20	120.00
O2—C18—C19	111.79 (13)	C20—C21—H21	120.00
C18—C19—C20	121.59 (16)	C22—C21—H21	120.00
C18—C19—C24	120.10 (15)	C21—C22—H22	120.00
C20—C19—C24	118.26 (17)	C23—C22—H22	120.00
C19—C20—C21	120.34 (17)	C22—C23—H23	120.00
C20—C21—C22	120.71 (17)	C24—C23—H23	120.00
C21—C22—C23	119.32 (19)	C19—C24—H24	119.00
C22—C23—C24	119.91 (19)	C23—C24—H24	119.00
			
C18—O2—C15—C14	18.0 (2)	C1—C9—C10—N1	178.76 (15)
C18—O2—C15—C16	−163.17 (15)	N1—C11—C12—C13	2.9 (3)
C15—O2—C18—C19	72.73 (18)	N1—C11—C12—C17	−175.88 (18)
C11—N1—C10—C9	−96.7 (2)	C11—C12—C13—O1	0.7 (3)
C10—N1—C11—C12	177.96 (17)	C11—C12—C13—C14	−179.48 (16)
C8—N2—C2—C1	0.1 (2)	C17—C12—C13—O1	179.48 (16)
C2—N2—C8—C3	178.73 (18)	C17—C12—C13—C14	−0.7 (2)
C2—N2—C8—C7	−0.4 (2)	C11—C12—C17—C16	177.17 (18)
C7—C1—C2—N2	0.3 (2)	C13—C12—C17—C16	−1.6 (3)
C9—C1—C2—N2	−177.68 (17)	O1—C13—C14—C15	−177.50 (16)
C2—C1—C7—C6	179.80 (18)	C12—C13—C14—C15	2.7 (2)
C2—C1—C7—C8	−0.48 (19)	C13—C14—C15—O2	176.31 (15)
C9—C1—C7—C6	−2.2 (3)	C13—C14—C15—C16	−2.4 (3)
C9—C1—C7—C8	177.52 (16)	O2—C15—C16—C17	−178.82 (17)
C2—C1—C9—C10	20.0 (3)	C14—C15—C16—C17	0.0 (3)
C7—C1—C9—C10	−157.53 (17)	C15—C16—C17—C12	2.0 (3)
C8—C3—C4—C5	−0.5 (3)	O2—C18—C19—C20	27.2 (2)
C4—C3—C8—N2	−179.09 (19)	O2—C18—C19—C24	−155.53 (16)
C4—C3—C8—C7	−0.1 (3)	C18—C19—C20—C21	177.42 (17)
C3—C4—C5—C6	0.1 (3)	C24—C19—C20—C21	0.1 (3)
C4—C5—C6—C7	0.9 (3)	C18—C19—C24—C23	−176.71 (17)
C5—C6—C7—C1	178.23 (18)	C20—C19—C24—C23	0.7 (3)
C5—C6—C7—C8	−1.5 (2)	C19—C20—C21—C22	−1.1 (3)
C1—C7—C8—N2	0.52 (19)	C20—C21—C22—C23	1.4 (3)
C1—C7—C8—C3	−178.67 (16)	C21—C22—C23—C24	−0.7 (3)
C6—C7—C8—N2	−179.71 (15)	C22—C23—C24—C19	−0.4 (3)
C6—C7—C8—C3	1.1 (2)		

### (E)-5-Benzyloxy-2-({[2-(1H-indol-3-yl)ethyl]iminiumyl}methyl)phenolate (II) Hydrogen-bond geometry (Å, º)

Cg1, Cg2 and Cg4 are the centroids of rings N2/C1/C2/C7/C8, C3–C8 and C19–C24, respectively.

**Table:**

*D*—H···*A*	*D*—H	H···*A*	*D*···*A*	*D*—H···*A*
N1—H1*N*···O1	1.07 (3)	1.81 (3)	2.657 (2)	133 (2)
N1—H1*N*···O1^i^	1.07 (3)	2.19 (3)	3.004 (2)	131 (2)
C2—H2···O1^ii^	0.93	2.55	3.467 (2)	167
C23—H23···*Cg*2^i^	0.93	2.95	3.716 (2)	141
C24—H24···*Cg*1^i^	0.93	2.70	3.465 (3)	140
N2—H2*N*···*Cg*4^ii^	0.85 (2)	3.03 (2)	3.75 (3)	145 (2)

Symmetry codes: (i) −*x*+1, −*y*, −*z*; (ii) −*x*+2, −*y*, −*z*.
